# Complete plastome sequence of *Phalaenopsis cornu-cervi* (Vandeae, Orchidaceae)

**DOI:** 10.1080/23802359.2019.1674720

**Published:** 2019-10-16

**Authors:** Jie-Yu Wang, Guo-Qiang Zhang, Chang-Cao Peng

**Affiliations:** a State Key Laboratory for Conservation and Utilization of Subtropical Agro-bioresources, South China Agricultural University, Guangzhou, China;; b Guangdong Key Laboratory for Innovative Development and Utilization of Forest Plant Germplasm, College of Forestry and Landscape Architecture, South China Agricultural University, Guangzhou, China;; c Shenzhen Key Laboratory for Orchid Conservation and Utilization, The National Orchid Conservation Centre of China and The Orchid Conservation and Research Centre of Shenzhen, Shenzhen, China

**Keywords:** Section Polychilos, *Phalaenopsis cornu-cervi*, chloroplast genome

## Abstract

*Phalaenopsis cornu-cervi* is a taxonomically and horticulturally important moth orchid. In this study, we report and characterize the complete plastid genome sequence of *P. cornu-cervi* for the first genomic resources in section *Polychilos*. Its complete plastome is 147,241 bp in length and contains two inverted repeat (IR) regions of 25,005bp, a large single-copy (LSC) region of 85,714 bp, and a small single-copy (SSC) region of 11,517 bp. The plastome contains 110 genes, consisting of 76 unique protein-coding genes, 30 unique tRNA genes, and 4 unique rRNA genes. It also shows the typical characteristics of *Phalaenopsis* chloroplast genome, while all *ndh* genes are non-functional. The complete plastome sequence of *P. cornu-cervi* will provide a useful resource for future phylogenetic study of *Phalaenopsis* and its garden utilization.

Commonly referred to as the ‘moth orchid’, *Phalaenopsis* is one of the well-known genera in Orchidaceae that occupied a large proportion of ornamental market shares (Van Huylenbroeck 2018). In this genus, subgenus *Phalaenopsis* has the showy flower and also widely applied as the crossing parents for breeding within moth orchids or between close genera. Subgenus *Phalaenopsis* was classified into two sections, sect. *Phalaenopsis* and sect. *Polychilos*, three chloroplast genomes of section *Phalaenopsis* have been published (Chang et al. 2006; Jheng et al. 2012; Kim et al. 2016), however, no genetic data of species belonging to sect. *Polychilos* is obtained yet. Thus, we obtained the plastome of *P. cornu-cervi* (GenBank accession number: MN395041), the type species of sect. *Polychilos*, as the sequenced target to provide genetic and genomic information for this section to promote the systematics research and garden utilization of *Phalaenopsis*.

*Phalaenopsis cornu-cervi* was sampled from National Orchid Conservation Centre in Guangdong province of China (114°19'01''E, 22°60'34'' N). A voucher specimen (*Noccphal052*) was deposited in the Herbarium of National Orchid Conservation Centre, Shenzhen, China. DNA was acquired from the young leaves of its plant and the total genome was sequenced using Illumina HiSeq 2000 platform. Then, the plastid source reads were identified by mapping them against the published *Phalaenopsis* chloroplast genomes by BLAST. Later, we applied PLATANUS to conduct the assembly with the modification by Geneious 2019.0.3. Geneious 2019.0.3 and online pipeline Geseq (Tillich et al. 2017) were used to complete the annotation against the plastome of *Phalaenopsis aphrodite* var. *formosa*. We used the BLAST to confirm the IR boundaries for the draft chloroplast genome.

The total chloroplast genome size of *P. cornu-cervi* is 147,241 bp, including a large single-copy (LSC) region (85,714 bp), a small single-copy region (11,517 bp), and two inverted repeat (IRs) regions (25,005 bp). The overall GC contents of the plastid genome were 36.7%. In total, the plastome contains 110 unique genes, consisting of 76 protein-coding genes, 4 rRNA genes, and 30 tRNA genes. The *ndh* genes were also non-functional in *P. cornu-cervi*, and *ndhA*, *ndhF*, and *ndhH* genes were completely absent as previous description by Chang et al. (2006). Moreover, the *ycf3*, *ycf1*, *rps16*, *rpoC2* and *atpF* were also non-functional in *P. cornu-cervi*, indicating the unique plastome features of sect. *Polychilos* species.

We used RAxML (Stamatakis 2006) with 1000 bootstraps under the GTRGAMMAI substitution model to reconstruct a maximum likelihood (ML) phylogeny of *P. cornu-cervi* and 13 published complete plastomes of Orchidaceae, using *Allium cepa* (Amaryllidaceae) as outgroups. The phylogenetic analysis indicated that *P. cornu-cervi* was situated as the sister of sect. *Phalaenopsis* clade (Figure 1). The complete plastome sequence of *P. cornu-cervi* will provide useful information for phylogenetic studies and future breeding in *Phalaenopsis* even in Orchidaceae.

**Figure 1.**
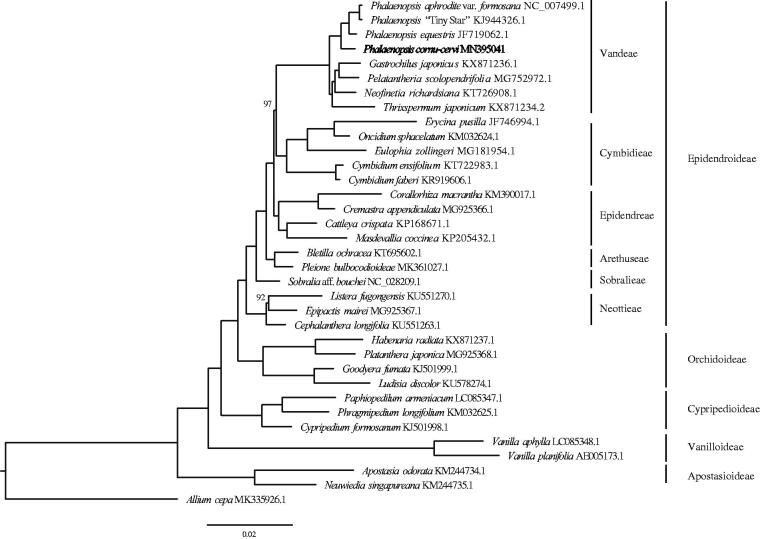
Maximum-likelihood phylogenetic tree reconstructed by RAxML based on complete plastome sequences from *P. cornu-cervi*, and thirty-three other orchids representing different tribes and subfamilies with *Allium cepa* as outgroup (Amaryllidaceae). Numbers on branches are bootstrap support values below than 100.
